# Trauma exposure and refugee status as predictors of mental health outcomes in treatment-seeking refugees

**DOI:** 10.1192/pb.bp.114.047951

**Published:** 2015-08

**Authors:** Jeroen W. Knipscheer, Marieke Sleijpen, Trudy Mooren, F. Jackie June ter Heide, Niels van der Aa

**Affiliations:** 1 Arq Psychotrauma Expert Group, Diemen/Oegstgeest, The Netherlands; 2 Utrecht University, Department of Clinical and Health Psychology, Utrecht, The Netherlands

## Abstract

**Aims and method** This study aimed to identify predictors of symptom severity for post-traumatic stress disorder (PTSD) and depression in asylum seekers and refugees referred to a specialised mental health centre. Trauma exposure (number and domain of event), refugee status and severity of PTSD and depression were assessed in 688 refugees.

**Results** Symptom severity of PTSD and depression was significantly associated with lack of refugee status and accumulation of traumatic events. Four domains of traumatic events (human rights abuse, lack of necessities, traumatic loss, and separation from others) were not uniquely associated with symptom severity. All factors taken together explained 11% of variance in PTSD and depression.

**Clinical implications** To account for multiple predictors of symptom severity including multiple traumatic events, treatment for traumatised refugees may need to be multimodal and enable the processing of multiple traumatic memories within a reasonable time-frame.

Refugees and asylum seekers have been shown to be at substantially higher risk of developing post-traumatic stress disorder (PTSD)^1^ and comorbid mental health problems than the general population,^2^ compatriots who have stayed in the refugees' home country,^3^ and economic migrants.^4^ Exposure to a high number of potentially traumatic events, involvement in asylum-seeking procedures and forced migration are stressors that set refugees apart from other populations and increase their psychological vulnerability. In Western psychiatric practice, insight in the determinants of PTSD and comorbid disorders in refugees may be helpful in guiding treatment interventions. Meta-analyses have shown that torture and cumulative traumatic events are the main predictors for development of PTSD in refugees.^5^ Additionally, researchers have begun to investigate whether different symptom profiles in refugees are related to different traumatic experiences. In a sample of Bosnian refugees, Momartin *et al*^6^ found that life threat and traumatic loss were related to PTSD and depression. In a sample of Mandean refugees, Nickerson *et al*^7^ found that PTSD and prolonged grief disorder were predicted by traumatic loss, whereas PTSD only was predicted by difficulties related to loss of culture and support.

While these findings are highly relevant to treatment-seeking refugees, similar analyses have not yet been carried out in large, treatment-seeking refugee samples. In this study we examine whether symptom severity of PTSD and depression is predicted by refugee status as well as accumulation and specific domains of traumatic events in a sample of refugees seeking specialised mental healthcare.

## Method

### Participants

Participants were asylum seekers and refugees referred for treatment at Foundation Centrum '45, a specialised Dutch centre for diagnosis and treatment of complex psychological trauma. Refugees are individuals who have been granted temporary or permanent refugee status in The Netherlands whereas asylum seekers are those still awaiting a final decision. In 2001, Foundation Centrum '45 started to routinely monitor treatment outcomes by administering questionnaires to patients at intake and annually during treatment. For the present study, data collected at intake were used. Complete data with regard to refugee status, traumatic experiences and symptom severity of PTSD and depression were available for 688 asylum seekers and refugees. Characteristics for the total sample are presented in Table 1. Participants came from three main regions: 58% from the Middle East (e.g. Afghanistan, Iraq, Egypt, Libya), 17% from Sub-Saharan Africa (e.g. Burkina Faso, Eritrea, Congo) and 16% from Balkan Europe (Bosnia, former Yugoslavia, Croatia).

**Table 1:** Demographic characteristics of the total sample and the main regions of origin

	Total sample (*n* = 688)	Middle East (*n* = 397)	Sub-Saharan Africa (*n* = 114)	Balkan Europe (*n* = 111)	Other regions (*n* = 66)
Male, *n* (%)	488 (71)	306 (77)	66 (58)	70 (63)	45 (68)

Age, mean (s.d.)	40.4 (10.4)	41.3 (9.9)	32.2 (8.5)	45.2 (9.1)	41.4 (11.2)

Permanent refugee status, *n* (%)	585 (85)	361 (91)	73 (64)	107 (96)	44 (67)

### Measures

Traumatic experiences and PTSD symptom severity were assessed using the Harvard Trauma Questionnaire (HTQ).^8^ The HTQ consists of three parts, the first two of which were used in this study. In the first part participants were asked to indicate which of the 19 traumatic events they experienced, witnessed or heard of, and only self-experienced traumatic events were used in the study. For each participant a total number of different self-experienced traumatic events was calculated (range 0-19). In the second part of the HTQ the severity of DSM-IV PTSD symptoms was assessed by asking participants how much they were bothered by 16 PTSD symptoms during the past week, rated on a 4-point scale ranging from 1, not at all to 4, extremely. PTSD symptom severity was computed by averaging responses (range 1-4). The HTQ recommends a cut-off score of 2.5 to identify clinically significant PTSD. Internal consistency of the scale was high (Cronbach's α 0.86).

Symptom severity for depression was assessed with the Hopkins Symptom Checklist (HSCL-25).^9^ Participants were asked to indicate how distressed they were by rating 10 symptoms of anxiety and 15 symptoms of depression during the past week on a 4-point scale ranging from 1, not at all to 4, extremely. Symptom severity of depression (range 1-4) was computed by averaging responses on the 15 depression items. The HSCL-25 recommends a cut-off score of 1.75 to indicate clinically significant depression. Internal consistency was high (Cronbach's α 0.89).

The HTQ and HSCL-25 are self-report questionnaires that are widely used with refugees and are available in many different languages. Questionnaires were administered in the patient's native language if possible and interpreters were used when necessary. Both instruments have good psychometric properties.^10^

### Statistical analyses

Statistical analyses were performed using SPSS version 20. To investigate whether specific domains of traumatic events could be identified, a principal component analysis with oblique rotation (direct oblimin) was conducted on the total set of self-experienced traumatic events. An initial analysis was run to obtain eigenvalues for each factor in the data and to evaluate the substantive contribution of each item to the extracted factors. Stevens^11^ recommends interpreting factor loadings greater than 0.4 as substantive. The analysis was rerun without the items that did not contribute substantively to the extracted factors. For each participant a total score was computed on each of the extracted domains of traumatic events by counting the total number of self-experienced traumatic events within the domain.

Hierarchical regression analyses were used to test whether refugee status, total number of self-experienced traumatic events and the domains of traumatic events predicted symptom severity of PTSD and depression. These variables were independently added to the regression models, together with the gender and age covariates, as these have been found to predict PTSD in refugee samples.^12^ Scores on the extracted domains of traumatic events were recoded into dummy variables before being added to the hierarchical regression models. To allow for multiple tests the alpha level of significance was set to 0.01.

## Results

First, it was investigated whether specific domains of traumatic events could be identified by conducting a principal component analysis. In the initial analysis, four factors had eigenvalues greater than 1. Four traumatic events (combat situation, brainwashing, rape or sexual abuse, and being close to death) did not contribute substantively to any of the four extracted factors and the analysis was rerun without these items. Four factors were retained with eigenvalues greater than 1.0, which together accounted for 56.7% of the total variance. Table 2 presents the factor loadings after rotation. The traumatic events that cluster on the same factor suggest that the first factor represents human rights abuses (31.1% of the total variation), the second factor traumatic loss (10.0% of the total variation), the third factor a lack of necessities (8.7% of the total variation), and the fourth factor separation from others (6.9% of the total variation).

**Table 2:** Summary of principal component analysis for traumatic experiences^a^

	Rotated factor loadings			
	Human rights abuses	Traumatic loss	Lack of basic human needs	Separation from others
Threatened to be executed	**0.78**	0.13	−0.13	0.09

Physical torture	**0.77**	−0.10	−0.03	−0.12

Threatened to be physically tortured	**0.77**	−0.03	−0.10	−0.08

Threatened to watch torturing	**0.66**	0.04	0.07	−0.04

Serious injury	**0.50**	0.01	0.21	0.11

Lost or kidnapped	**0.46**	0.15	0.13	−0.04

Imprisonment	**0.46**	−0.04	0.16	−0.28

Murder of family or friend	0.02	**0.85**	−0.04	0.01

Unnatural death of family or friend	−0.12	**0.79**	0.04	−0.11

Murder of stranger or strangers	0.15	**0.63**	0.03	0.02

Lack of shelter	−0.03	0.05	**0.79**	0.12

Lack of food or water	−0.07	0.03	**0.78**	−0.15

Ill health without access to medical care	0.13	−0.03	**0.68**	−0.07

Forced separation from family members	−0.04	0.09	−0.02	−0.85

Forced isolation from others	0.14	0.03	0.06	−0.75

a. Factor loadings over 0.40 appear in bold.

Mean symptom severity was 3.1 (s.d. 0.5) for PTSD and 2.9 (s.d. 0.6) for depression both within the clinical range. A clinical level of symptom severity for PTSD and depression was reported by, respectively, 84% and 95% of participants. Participants reported a mean of 11.2 different self-experienced traumatic events (s.d. 4.7). The most commonly reported events were being close to death (80%), forced separation from family members (74%), murder of family or friend (72%), threatened to be physically tortured (72%), and unnatural death of family or friend (66%). With regard to the trauma domains, human rights abuses were reported by 90% of participants, traumatic losses by 83%, lack of necessities by 77%, and separation from others by 81%.

Hierarchical regression analyses were used to test whether possession of refugee status, the total number of different self-experienced traumatic events, and different domains of self-experienced traumatic events predicted symptom severity of PTSD and depression. Results of the hierarchical regression models are shown in Table 3. First, symptom severity of PTSD and depression was adjusted for gender and age by adding them to the model in step 1. Refugee status was added to the model in step 2. Lack of refugee status was significantly associated with increased symptom severity for PTSD and depression. Refugee status accounted for 2% of the variation in symptom severity of those disorders. Total number of different self-experienced traumatic events was added to the model in step 3. Increased number of different self-experienced traumatic events was significantly associated with increased symptom severity for PTSD and depression. Total number of different self-experienced traumatic events accounted for 8% of the variation in PTSD symptom severity and for 7% of the variation in symptom severity for depression. To test the unique effect of different domains of self-experienced traumatic events to symptom severity of PTSD and depression, human rights abuses, traumatic loss, lack of necessities and separation from others were added to the model in step 4. None of these domains were significantly associated with symptom severity. Adding the different domains of self-experienced traumatic events to the model accounted for an additional 1% of the variation in symptom severity.

**Table 3:** Hierarchical regression models of predictors of symptom severity with regard to PTSD and depression^a^

	PTSD symptoms				Depressive symptoms			
	B	s.e.	Beta	Δ*R*^2^	B	s.e.	Beta	Δ*R*^2^
Step 1				0.00				0.01
Constant	3.05	0.03			2.88	0.03		
Gender	0.04	0.05	0.03		0.11	0.05	0.08	
Age	0.02	0.02	0.03		0.03	0.02	0.05	

Step 2				0.02*				0.02*
Constant	3.02	0.03			2.84	0.03		
Gender	0.04	0.05	0.04		0.12	0.05	0.09	
Age	0.04	0.02	0.07		0.06	0.02	0.09	
Refugee status	0.21	0.06	0.14*		0.28	0.07	0.16*	

Step 3				0.08*				0.07*
Constant	3.00	0.03			2.82	0.03		
Gender	0.09	0.04	0.08		0.18	0.05	0.13*	
Age	0.04	0.02	0.07		0.06	0.02	0.09	
Refugee status	0.20	0.06	0.13*		0.26	0.07	0.15*	
Traumatic experiences, *n*	0.15	0.02	0.28*		0.17	0.02	0.28*	

Step 4				0.01				0.01
Constant	2.82	0.11			2.58	0.13		
Gender	0.09	0.04	0.08		0.17	0.05	0.12*	
Age	0.04	0.02	0.07		0.06	0.02	0.09	
Refugee status	0.20	0.06	0.13*		0.26	0.07	0.15*	
Traumatic experiences, *n*	0.11	0.03	0.21*		0.12	0.04	0.18*	
Human right abuses	0.15	0.08	0.09		0.09	0.09	0.05	
Traumatic loss	0.01	0.06	0.00		0.07	0.07	0.04	
Lack of basic human needs	0.05	0.06	0.04		0.04	0.07	0.03	
Separation from others	0.01	0.06	0.01		0.09	0.07	0.06	

B, Unstandardised regression coefficient; Beta, standardised regression coefficient; PTSD, post-traumatic stress disorder.

a. Dependent variables: symptom severity with regard to PTSD and depression.

* *P*<0.01

Δ*R*^2^, change in *R*^2^ compared with previous step.

### Clinical implications

In a large sample of asylum-seeking and refugee patients seeking treatment within a specialised Western mental health setting, PTSD symptom severity and depression was predicted by lack of refugee status and cumulative traumatic events, but not by specific domains of traumatic experience. Refugee status, total number of self-experienced traumatic events, domains of traumatic experiences, and gender and age together accounted for only 11% of variation in symptom severity of PTSD and depression. These results are in stark contrast with earlier findings among non-treatment-seeking refugee populations, which showed that torture and cumulative traumatic events accounted for 34.4% of variance in PTSD prevalence rates and for 33.4% of variance in depression prevalence rates.^5^

Clearly, PTSD symptom and depression severity among asylum seekers and refugees seeking specialised treatment is influenced by multiple factors, including some that were not measured in this study. To map predictors for PTSD and depression in refugee patients, assessment may need to focus on a broader range of both stressors and resources, including stressors and resources related to forced migration, such as safety of family in the home country and social support. Rather than traumatic stress, it may be the burden of current stress and lack of resources that leads to PTSD and depression, prompting refugees to seek mental healthcare. This finding implies that clinically, in this severely traumatised population, an exclusive focus on processing of traumatic experiences as prescribed in PTSD treatment guidelines may result in only limited symptom reduction. Consequently, for refugee patients with severe psychopathology treatment may need to be multimodal rather than trauma-focused only.

In addition, in contrast to other studies, which showed an association between life threat and traumatic loss on the one hand and PTSD and comorbid disorders on the other, in our treatment-seeking sample no such associations were found. In our sample, the number rather than domain of traumatic events was associated with symptom severity. This implies that trauma-focused treatments for refugees should be designed to enable the processing of a large number of traumatic events within a reasonable time-frame. Treatments such as narrative exposure therapy and trauma-focused cognitive therapy may enable that to a greater extent than *in vitro* exposure therapy or eye movement desensitisation and reprocessing therapy, which in refugees may require several sessions for the processing of a single memory.

Although our findings can be generalised to mental healthcare-seeking refugees and asylum seekers only and the range of questionnaires was limited, merits of this study lie in the satisfactory cultural validity of the questionnaires and the large sample size. Future studies using a broader range of instruments are needed to identify predictors for PTSD and depression in treatment-seeking refugees.
